# Diallyl Trisulfide Attenuates Ischemia-Reperfusion-Induced ER Stress and Kidney Dysfunction in Aged Female Mice

**DOI:** 10.3390/cells14060420

**Published:** 2025-03-12

**Authors:** Sathnur Pushpakumar, Subir Kumar Juin, Hebah Almarshood, Dibson Dibe Gondim, Rosemary Ouseph, Utpal Sen

**Affiliations:** 1 Department of Physiology, University of Louisville School of Medicine, Louisville, KY 40202, USA; 2 Department of Microbiology and Immunology, University of Louisville School of Medicine, Louisville, KY 40202, USA; subirkumar.juin@louisville.edu; 3 Department of Pathology, University of Louisville School of Medicine, Louisville, KY 40202, USA; 4 Division of Nephrology & Hypertension, University of Louisville School of Medicine, Louisville, KY 40202, USA

**Keywords:** ischemia-reperfusion, kidney injury, ER stress, angiogenesis, H_2_S, signaling

## Abstract

Ischemia-reperfusion injury (IRI) is a common cause of acute kidney injury (AKI) in the aging population. Gender studies show that aging is associated with loss of protection from AKI in the female population. While ER stress contributes to IRI-induced AKI in the young, ER regulation during IR in the aged kidney is unclear. Because current evidence suggests hydrogen sulfide (H_2_S) modulates ER stress, we investigated whether exogenous supplementation of diallyl trisulfide (DATS), an H_2_S donor, mitigates AKI in aged female kidneys. Wild-type (WT, C57BL/6J) mice aged 75–78 weeks were treated with or without DATS before and after renal IRI. IRI increased ER stress proteins, inflammation, and fibrosis markers in the IRI kidney compared to the control. DATS mitigated ER stress, and reduced inflammation and fibrosis markers in the IRI kidney. Further, IRI kidneys demonstrated reduced blood flow, vascularity, angiogenesis, increased resistive index (RI), and reduced function. DATS treatment upregulated PI3K, AKT, p-mTOR, and pMAPK signaling to stimulate angiogenesis, which improved vascular density, blood flow, and renal function. Together, our results suggest that DATS rescues the aged female kidney IRI by modulating ER stress and upregulation of angiogenesis.

## 1. Introduction

Ischemia-reperfusion injury (IRI) is the leading cause of acute kidney injury (AKI) in the perioperative period and is associated with increased morbidity and mortality [1,2]. Renal IRI induces several structural and functional alterations involving glomerular and vascular endothelial cells and tubular epithelial cells that together disrupt renal function [3]. Recent studies suggest endoplasmic reticulum (ER) plays an important role in renal IRI [4]. ER is a dynamic intracellular organ system involved in protein synthesis, post-translational modification, folding and transport, Ca^2+^ handling, and signal transduction [5,6]. Under physiological conditions, the unfolded protein response (UPR) system maintains a balance between the protein load and protein folding capacity in the ER, whereas in pathophysiology, the fine balance is disrupted leading to the activation of three ER transmembrane sensors, (1) protein kinase RNA-like ER eukaryotic initiation factor-2α kinase (PERK), (2) inositol requiring protein 1 (IRE1), and (3) activating transcription factor 6 (ATF6), that attenuate protein translation to mitigate ER stress [7]. A consequence of PERK activation is the induction and translocation of NF-kB to the nucleus where it triggers proinflammatory genes [8]. During renal IRI, the accumulation of unfolded and misfolded proteins causes ER stress and disruption of a network of mechanisms to determine cell fate [9,10]. While mild to moderate ER stress is cytoprotective, excess ER stress impairs protein homeostasis and triggers apoptotic pathways [4,11].

The renal microvasculature plays an important role in repair and regeneration following IRI. As a primary barrier to insults, the endothelial cells sustain damage during ischemia and reperfusion phases triggering a cascade of events leading to stress, inflammation, and cytoskeletal derangements [12]. Together, the events cause endothelial activation and dysfunction characterized by compromised vascular tone, and loss of capillary density that are crucial for the recovery process [13,14,15]. The renal outer medulla is sensitive to hypoxia and loss of peritubular capillaries decreases perfusion, worsening injury and delaying repair [16]. The downregulation of angiogenic factor, vascular endothelial growth factor-A (VEGF-A) is implicated in capillary rarefaction and reduced angiogenesis following IRI [17,18]. Interestingly, VEGF-A expression by the tubular epithelial cells plays an important role in maintaining tubular–endothelial cell crosstalk that directly affects endothelial function [19,20]. Several studies demonstrated that ER stress mediates endothelial dysfunction in diabetes [21], hypertension [22], hyperhomocysteinemia [23], and hyperlipidemia [24] involving various organ systems [25].

Hydrogen sulfide (H_2_S) is a gasotransmitter that is known to perform numerous functions in health and disease states. Previously, we demonstrated that in renal IRI, deficiency of H_2_S is associated with renal inflammation and fibrosis, and supplementation of H_2_S decreased macrophage polarization to inflammatory phenotype and endothelial-mesenchymal transition in the aged kidney [26]. Recent studies suggest that H_2_S modulates ER stress in cardiovascular pathology [27,28], neurological disorders [29,30], and vascular disease [31].

The purpose of the present study was to investigate whether diallyl trisulfide (DATS), a H_2_S donor, offers renal protection from IRI-induced ER stress and to test whether enhancing angiogenesis reverses loss of medullary blood flow and renal dysfunction.

## 2. Materials and Methods

### 2.1. Animal Groups and DATS Treatment

C57BL/6J wild type (WT, Stock no.: 000664) mice were purchased from Jackson Laboratory (Bar Harbor, ME, USA) and bred in-house, housed in a temperature- and light-controlled environment at the University of Louisville animal facility. Female mice aged 75–78 weeks were used in the study. The animals were randomized (n = 6/group, total n = 24) into the following groups: (1) WT mice fed a normal diet (WT+ND) that served as control group, (2) WT+ND+IRI, (3) WT+DATS, and (4) WT+DATS+IRI. The sample size and the experimental groups were determined to ensure a *p*-value of 0.05 or lower for the variables based on our prior study [26]. Additional mice were included in the study due to severity of injury and death. Human end point and sudden death accounted for <5% of the total number of mice, and data from these animals were excluded from analysis. The final analysis included all animals that survived until the end point of the study.

The animals were fed a customized diet containing diallyl trisulfide (DATS) for 21 days prior to IRI and continued for 7 days afterward. The DATS dosage was based on previous studies, with a formulation of 50 ppm, equivalent to 50 mg/kg/day [32,33]. This dosage calculation was based on the average daily food intake of approximately 4 g per mouse. Incorporating DATS into the chow allowed us to avoid complications such as internal injury and inflammation, as well as issues related to repeated intraperitoneal injections over the four-week study period. Controls were given a standard diet and tap water ad libitum. At the end of the experiments, the animals were euthanized using 2X tribromoethanol (TBE), and blood samples were collected in lithium heparin vacutainer tubes to separate plasma as described [34].

The animal protocols were performed in accordance with institutional animal care guidelines and conform to the Guide for the Care and Use of Laboratory Animals published by the U.S. National Institutes of Health (NIH Publication, 2011). The study was approved by the Institutional Animal Care and Use Committee (IACUC) of the University of Louisville School of Medicine (IACUC#21967, date: 28 September 2021).

### 2.2. Rationale for the Chosen Age Range

Generally, acute kidney injury (AKI) is statistically more prevalent in both female and male individuals over the age of 65 compared to those aged 18 to 65 years [35]. A recent study also reported higher rates of AKI among individuals in the median age range of 59 to 68 years for both females and males [36]. In animal studies, mice aged 18 to 24 months (approximately 72 to 96 weeks) are considered old, which closely resembles human aging between 56 to 69 years (The Jackson Laboratory, Bar Harbor, ME, USA). Moreover, there are fewer studies focusing on females compared to males. For these reasons, we used female mice aged 75 to 78 weeks in our AKI study to better simulate the corresponding human age group.

### 2.3. Ischemia-Reperfusion Injury (IRI)

Experimental renal IRI in the mouse replicates human acute kidney injury and is therefore widely used by researchers to study the IRI mechanisms and response to treatment. In the present study, renal IRI was created by a single researcher as described before with modification [26]. Briefly, mice were anesthetized by isoflurane inhalation (4% induction and 1% maintenance) and given preemptive meloxicam analgesia (10 mg/kg, SC). Through a midline dorsal incision, the renal vessels were exposed on both sides and microvascular clamps were applied for 27 min. The clamps were removed to allow reperfusion, and the wound closed in layers. The mice received analgesia for three consecutive days and postoperative care until the end point at 7 d post IRI. The analgesia dose/duration is nontoxic to mice kidneys [37,38]. Euthanasia and sample collection was carried out between 10 AM and 1 PM.

### 2.4. Antibodies and Reagents

ATF6 (Cat. No.: 66563-1-IG), Phospho-IRE1α (Cat. No.: PA5-105425, Thermo Fisher Scientific, Waltham, MA, USA), Phospho-PERK (Cat. No.: PA5-102853, Thermo Fisher Scientific), NF-kB p65 (Cat. No.: 51-0500), IL-17A (Cat. No.: PA5-106856), Col1A1 (Cat. No.: PA5-29565), phosphor-mTOR (Cat. No.: 44-1125G), anti-rabbit Alexa Fluor 488 (Cat. No.: A11008), and anti-rabbit Alexa fluor 594 (Cat. No.: A11012) were from Thermo Fisher Scientific. VEGF-A (Cat. No.: ab51745), CD31 (Cat. No.: ab9498), HIF1α (Cat. No.: ab82832), and TGF-β (Cat. No.: ab64715) were from Abcam, Cambridge, UK. TNF-α (Cat. No.: 60291-1-Ig, Proteintech, Rosemont, IL, USA), IL-1β (Cat. No.: AF-401-NA, Novus Biologicals, Centennial, CO, USA), Lipocalin-2 (Cat. No.: AF1857, R&D Systems, Minneapolis, MN, USA), eNOS (Cat. No.: 610297, BD Biosciences, Franklin Lakes, NJ, USA), p-PI3K (Cat. No.: 4228), p-AKT (Cat. No.: 4060), and p-MAPK (Cat. No.: 9211) were from Cell Signaling, Danvers, MA, USA. Β-actin (Cat. No.: SC47778) and Erythropoietin (sc-5290) were from Santa Cruz Biotechnology, Dallas, TX, USA. QuantiChrom^TM^ creatinine assay kit (Cat. No.: DICT-500) was from BioAssay Systems, Hayward, CA, USA. Manufacturer’s instructions were followed for all applications.

### 2.5. H_2_S Measurement

Renal H_2_S was measured as described before with modification [39]. Briefly, fresh kidney samples were washed and homogenized in ice-cold PBS. After centrifugation, the supernatant (100 μL) was mixed in a centrifuge tube containing PBS (100 mM, pH 7.4, 350 μL) and zinc acetate (1% W/V, 250 μL). This step was followed by the addition of N,N-dimethyl-p-phenylenediamine sulfate (20 mM, 133 μL) in 7.2 M HCl, and FeCl_3_ (30 mM, 133 μL) in 1.2 M HCl. The mixture was sealed tightly and incubated at 37 °C for 45 min. The reaction was terminated by adding trichloroacetic acid (TCA) solution (10% *W*/*V*, 250 μL). After centrifugation, 200 μL supernatant was transferred to a 96-well plate and absorbance was read at 670 nm using a spectrophotometer. Samples were assayed and H_2_S was calculated against a calibration curve of known concentrations of NaHS.

### 2.6. Antibody Authentication

All antibodies on Western blot were characterized by neutralizing antigens. Control lane using the standard of protein was used in each blot. This established the authenticity of the antibody and was further used for other Western blots, immunohistochemistry, and immunostaining analyses. To reduce non-specificity, we incubated BSA blocked membrane with antibodies to deplete the non-specific binding of protein fractions.

### 2.7. Western Blotting

Proteins were extracted from whole kidney lysates and electrophoresed on SDS-PAGE and transferred to the PVDF membrane by following previous protocol [26,40]. After blocking, primary antibodies were incubated overnight at 4 °C. Appropriate secondary antibodies were incubated for 120 min at room temp. The membranes were developed using chemiluminescence and visualized with the ChemiDoc MP system (BioRad, Hercules, CA, USA). Band intensities were quantified using ImageJ software (https://imagej.net/ij/ (accessed on 2 April 2022).

### 2.8. Gene Expression

Total RNA was extracted using Quick-RNA^TM^ MiniPrep (Cat. No.: R1055, Zymo Research, Irvine, CA, USA) and cDNA was synthesized using EasyScript cDNA Synthesis Kit (Cat. No.: G234, MidSci, St. Louis, MO, USA) following manufacturer’s instructions. The mRNA levels were quantified by qPCR (Lightcycler^®^ 96 system, Roche Diagnostics Corporation, Indianapolis, IN, USA) using the specific primers listed in Table 1.

### 2.9. Immunohistochemistry

IHC was conducted as described before [26,40]. Frozen kidneys at 5 μm thickness were air-dried and fixed in acetone for 10 min. After blocking, sections were incubated with primary antibodies overnight at 4 °C. Immune labeling was conducted using Alexa fluor 488 and Alexa fluor 594 secondary antibodies for 90 min at room temperature. Images were captured using Olympus FluoView1000 (B&B Microscope Ltd., Pittsburgh, PA, USA). Mean fluorescence intensity was measured using ImageJ software (1.53q 30 March 2022), as stated earlier.

### 2.10. Renal Cortical Blood Flow

Renal cortical blood flow was measured after 7 days of IR using a Speckle Contrast Imager (Moor FLPI, Wilmington, DE, USA), as described before [40,41]. Briefly, a dorsal incision was made to expose the kidney, and the camera was focused on the renal cortex to obtain cortical flux units (No. of RBCs x velocity).

### 2.11. Renal Microvasculature

The renal microvasculature was visualized at the end point of experiment, i.e., after 7 days of IR using our previously adopted barium sulfate angiography [26]. Briefly, the abdomen was opened with a midline incision, and the left renal artery was exposed by microdissection. A PE10 (ID—0.28 mm, BD, Franklin Lakes, NJ, USA) was introduced via arteriotomy and a barium sulfate solution was perfused using automated syringe pump. Images were captured using a Kodak FX Pro in vivo imaging system (Molecular Imaging System; Carestream Health Inc., Rochester, NY, USA) and microvasculature density was analyzed using VesSeg software (VesSeg_V0.1.4; University of Lubeck, Lubeck, Germany).

### 2.12. Conventional Ultrasonography

After 7 days post IRI, conventional B-mode ultrasonography was conducted using Vevo 2100 (VisualSonics, Toronto, ON, Canada) as described before [26]. Briefly, mice were anesthetized using isoflurane, and the ventral abdominal area was depilated. After acoustic gel (Other-Sonic; Pharmaceutical Innovations, Newark, NJ, USA) application on the skin, an MS550D transducer (22–25 Mz) was used to image outer medullary vessels in the short axis to obtain peak systolic and end diastolic velocities. The data was exported to measure resistive index (RI) of the medullary microvessels using the formula described before [42].

### 2.13. Renal Function

Glomerular filtration rate (GFR) was used as surrogate to measure renal function in conscious mice [26,43]. GFR was measured 7 days after IR. Briefly, under isoflurane anesthesia, the left side of abdominal dorsum was depilated for the application of NIC-kidney device (MediBeacon, St. Louis, MO, USA). A solution of FITC-sinistrin (7 mg/100 g b.w., MediBeacon, St. Louis, MO, USA) was injected intravenously and data acquired for 2 h. The half-life of FITC-sinistrin was recorded using MediBeacon software (version: vol.1.1), and the GFR was calculated with a conversion factor as previously described [26,43].

Plasma creatinine was measured as described previously using QuantiChrom^TM^ creatinine assay kit [39]. Briefly, samples (30 μL) and STD (2 mg/dL, 30 μL) were transferred to a 96-well clear bottom plate. Working reagent (200 μL) was added to each well and mixed gently. The optical density was measured at 0 min and 5 min using a SpectraMax M2e (Molecular devices, San Jose, CA, USA) set at 510 nm. Plasma creatinine concentration was calculated using the following formula: [(OD sample 5 − ODsample 0)/(OD standard 5 − OD standard 0)] × [STD] (mg/dL).

### 2.14. Statistical Analysis and Blinding

Data are presented as mean ± SD, ‘n’ represents the number of animals used. The difference between the groups was determined by ANOVA followed by a post hoc Tukey test. A Mann–Whitney U test was conducted for nonparametric data. Differences with a *p*-value < 0.05 were considered significant. All data analysis was conducted using GraphPad Prism (10.1.2). The animals were assigned by randomization into their groups by the PI. All animals that survived until the end point were included for data analysis. Investigators who carried out tissue sample preparation, experiments, measurements, and analysis were blinded to the study groups.

## 3. Results

### 3.1. DATS Attenuated IRI-Induced ER Stress in the Aged Kidney

**Figure 1:**
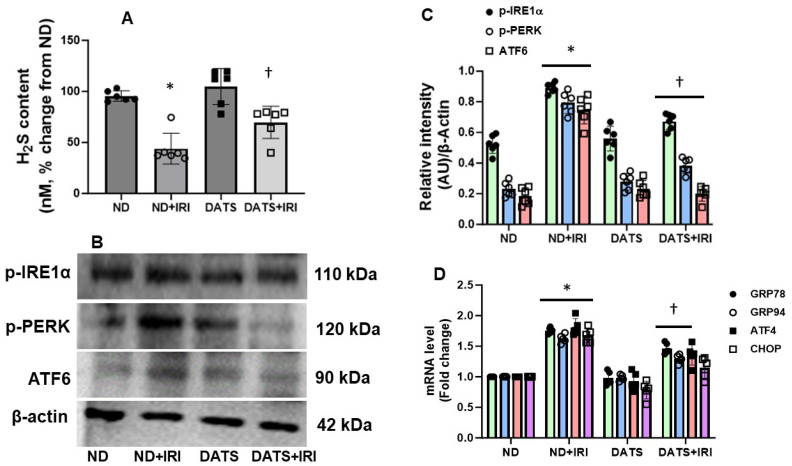
DATS attenuated ER stress in IRI of the aged kidney. (**A**) H_2_S content in the kidney. Values are expressed as mean ± SD, n = 6/group, * *p* < 0.01 vs. other groups, ^†^ *p* < 0.01 vs. ND+IRI. (B) Protein expression of phospho-inositol requiring enzyme 1 alpha (p-IRE1α), phosphor PRKR-like endoplasmic reticulum kinase (p-PERK), and activating transcription factor 6 (ATF6). (C) Densitometry analysis of protein bands normalized to β-actin, values are mean ± SD, n = 6/group, * *p* < 0.01 vs. ND, ^†^ *p* < 0.01 vs. ND+IRI. (D) Fold change of mRNA levels of ER stress markers, glucose-regulated protein 78 (GRP78), glucose-regulated protein (GRP94), activating transcription factor 4 (ATF4), and C/EBP homologous protein (CHOP). Values are expressed as mean ± SD, n = 6/group, * *p* < 0.05 vs. ND, ^†^ *p* < 0.01 vs. ND+IRI.

To determine changes in the H_2_S levels in the kidney and the response to IRI, we measured H_2_S by methylene blue method as described in the material and methods. The H_2_S levels in normal diet (ND) mice served as control. In the ND+IRI mice, renal H_2_S content was reduced compared to ND (Figure 1A). In the DATS+IRI mice, H_2_S content was increased significantly but did not normalize to the levels observed in the control group (Figure 1A). DATS alone treatment in the ND group showed an increasing trend but was not significant from the control ND group.

ER stress in the aged kidney was quantified by measuring changes in the expression of UPR membrane proteins, p-IRE1α, p-PERK, and ATF6. At 7 days post IRI, the expression of all three proteins was increased in normal diet (ND)-fed mice following IRI, compared to control mice fed ND (Figure 1B,C). In the DATS+IRI mice, the expression of p-IRE1α, p-PERK, and ATF6 was significantly mitigated compared to ND+IRI (Figure 1B,C). DATS alone treatment in the ND group did not show any changes in their expression compared to the control ND group (Figure 1B,C).

Additionally, in the post IRI kidneys, the mRNA levels of transcription factor, ATF4, and C/EBP homologous protein (CHOP) were upregulated (Figure 1D). The increased expression is coupled with p-PERK (Figure 1B,C) suggesting the predominance of PERK pathway in renal IRI. In IRI mice fed with the DATS diet, the mRNA expression levels of ATF4 and CHOP were decreased compared to ND+IRI (Figure 1B–D). The expression of ATF4 and CHOP mRNAs remained unchanged in ND and DATS alone.

During the initial phase of stress, ER resident chaperones are expressed to regulate protein folding and excess UPR activation. We therefore measured ER resident chaperones, GRP78 and GRP94, heat shock proteins. In IRI kidneys, the mRNA levels of GRP78 and GRP94 were increased compared to control mice and DATS supplementation attenuated their expression (Figure 1D). No significant changes in their expression were observed in DATS alone compared to ND (Figure 1D).

**Figure 2:**
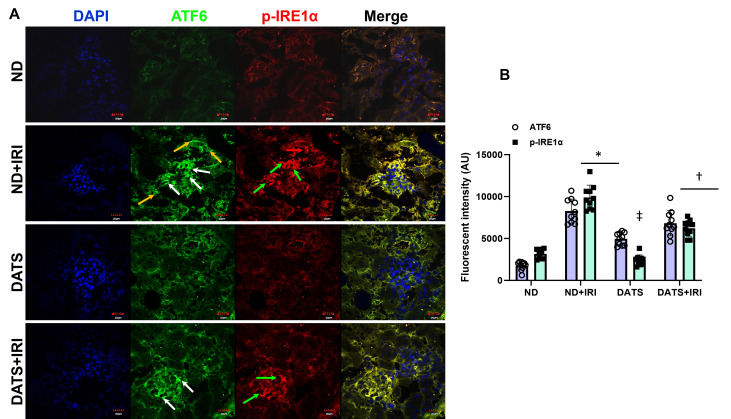
DATS treatment attenuated ER stress in aged kidney IRI. (**A**) Expression of unfolded protein response sensors, ATF6, and p-IRE1α. ATF6 was upregulated in the glomeruli and tubules (white and yellow arrows, respectively) and p-IRE1α was upregulated predominantly in the glomeruli compared to the tubules (green and red arrows, respectively). Scale bar, 20 μm. (**B**) Data are mean ± SD, n = 6/group, * *p* < 0.01 vs. ND, ^†^ *p* < 0.01 vs. ND and DATS, ^‡^ *p* < 0.01 vs. ND+IRI.

Immunohistochemical staining revealed p-IRE1α localized mainly to the glomeruli compared to tubules and ATF6 localized to both the glomeruli and tubular regions in IRI kidneys (Figure 2A). The p-IRE1α showed a 3-fold increase in fluorescent intensity and ATF6 increased by 4.7-fold compared to control mice, and their expressions were decreased in mice that received DATS treatment (Figure 2A,B). No significant changes in their expression were observed between ND and DATS alone (Figure 2A,B).

### 3.2. DATS Enhanced Renal Angiogenic Factors Following IRI

**Figure 3:**
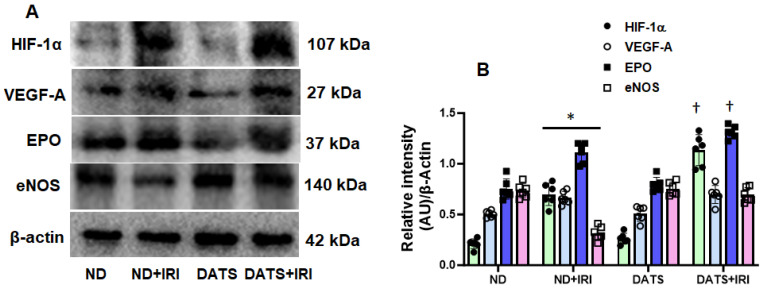
DATS supplementation in IRI enhanced the expression of angiogenesis markers in the kidney. (**A**) Representative bands of HIF-1α, VEGF-A, erythropoietin (EPO), and eNOS. (**B**) Densitometric analysis of protein expression as mean ± SD, n = 6/group, * *p* < 0.01 vs. ND or DATS alone, ^†^ *p* < 0.01 vs. ND+IRI.

Angiogenesis and vascular repair are crucial for recovery from IRI. To determine whether neo-angiogenesis re-establishes microcirculation in the kidney following IRI, we investigated the expression of angiogenic factors. In ND-fed mice, IRI increased the expression of hypoxia-inducible factor-1α (HIF-1α) along with VEGF-A and EPO; however, the eNOS expression was significantly decreased compared to control mice fed with ND and mice fed with DATS without IRI (Figure 3A,B). In DATS-fed mice that underwent IRI, VEGF-A expression remained unchanged, but the expression of HIF-1α, EPO, and eNOS was increased compared to IRI mice fed with ND (Figure 3A,B). DATS treatment alone did not alter the expression of HIF-1α, VEGF-A, EPO, and eNOS compared to ND-treated control mice (Figure 3A,B).

**Figure 4:**
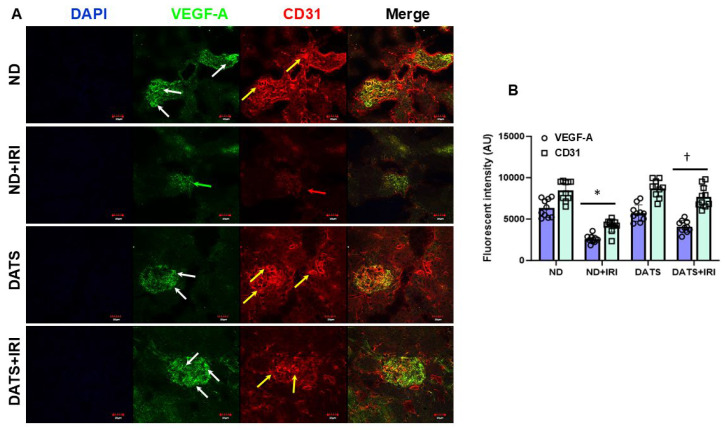
DATS treatment increased angiogenesis after IRI in the aged kidney. (**A**) Expression of angiogenesis markers, VEGF-A, and CD31 was decreased in the ND+IRI kidney (green and red arrows, respectively) and increased with DATS (white and yellow arrows, respectively). Scale bar, 20 μm (**B**) Data are mean ± SD, n = 6/group, * *p* < 0.01 vs. ND, ^†^ *p* < 0.01 vs. ND+IRI.

We further examined for angiogenesis markers, VEGF-A and CD31 in kidney sections by immunohistochemistry. In ND and DATS-fed mice that did not undergo IRI, VEGF-A and CD31 expression were similar in the glomeruli and peritubular areas (Figure 4A,B). In ND+IRI mice, both markers were decreased in the cortex and medullary regions (Figure 4A,B). In the DATS+IRI kidney, VEGF-A and CD31 expression were increased in both glomerular and peritubular areas (Figure 4A,B).

### 3.3. DATS Activated PI3K/AKT/mTOR Pathway to Induce Angiogenesis in Renal IRI

**Figure 5:**
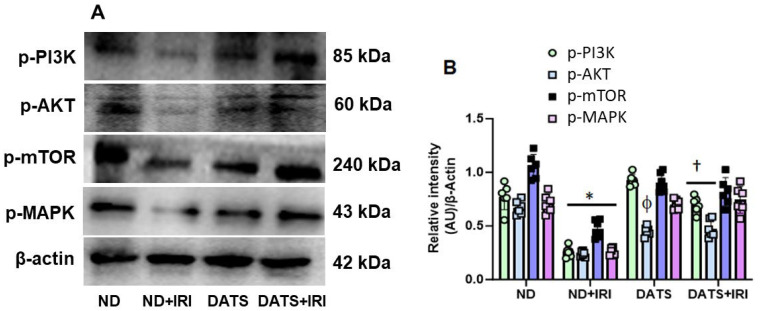
DATS activated p-PI3K, p-AKT, p-mTOR, and p-MAPK pathways in renal IRI. (**A**) Representative image of protein expression of signaling molecules of endothelial proliferation and survival. (**B**) Densitometry analysis of protein expression as mean ± SD, n = 6/group, *, ^ϕ^ *p* < 0.01 vs. ND, ^†^ *p* < 0.05 vs. ND+IRI.

Hypoxia is a major stimulus for HIF-1α induction that, in turn, leads to VEGF-A production by the podocytes and tubular epithelial cells in the kidney [44]. Additionally, VEGF-A-mediated stimulation of endothelial cells causes activation of the PI3K/AKT/mTOR pathway that results in angiogenesis [45]. To investigate whether H_2_S-induced angiogenesis occurs via activation of the PI3K pathway, we quantified the expression of signaling molecules. In the ND+IRI kidney, protein expression of p-PI3K, p-AKT, and p-mTOR was downregulated compared to the ND control kidney (Figure 5A,B). The p-PI3K expression was decreased by 3-fold, p-AKT by 2.8-fold, and p-mTOR by 2.2-fold compared to ND kidney without IRI. In the DATS+IRI kidney, the levels of p-PI3K, p-AKT, and p-mTOR were significantly increased and were similar to the levels observed in the ND kidney without IRI (Figure 5A,B). Early stages of angiogenesis require MAPK signaling for cell proliferation and differentiation [46]. We therefore measured the expression of phosphorylated-MAPK (p-MAPK). In the ND+IRI kidney, p-MAPK (p-MAPK) was significantly decreased compared to the ND kidney without IRI. Pre-treatment with DATS followed by IRI showed upregulation of p-MAPK compared to ND+IRI kidney (Figure 5A,B).

### 3.4. DATS Attenuated Renal Inflammation and Injury in the Aged Kidney IRI

**Figure 6:**
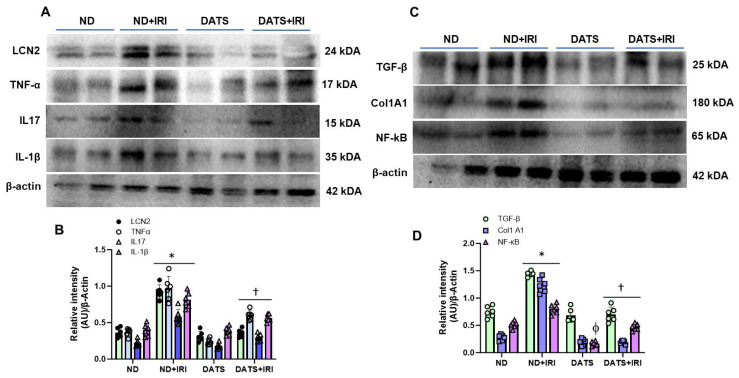
DATS treatment suppressed IRI-induced renal inflammation and fibrosis markers in the aged kidney. (**A**) Representative bands showing expression of renal injury marker, LCN2 (lipocalin 2), and inflammatory markers TNF-α, IL17, and IL-1β. (**B**) Data are mean ± SD, n = 6/group, * *p* < 0.01 vs. ND, ^†^ *p* < 0.01 vs. ND+IRI. (**C**) Representative bands for TGF-β, Col1A1, and NF-kB. (**D**) Data are mean ± SD, n = 6/group, * *p* < 0.01 vs. ND, ^†^, ^ϕ^ *p* < 0.01 vs. ND+IRI.

While sexual dimorphism in renal IRI is well known [47,48], the changes in IRI-induced inflammation in aged female kidney remains less studied. We therefore measured the protein expression of inflammatory markers, lipocalin-2 (LCN2), TNF-α, IL-17, and IL-1β. In the ND+IRI kidney, LCN2 increased by 2.5-fold, TNF-α by 2.6-fold, IL-17 by 2.6-fold, and IL-1β by 2-fold compared to the ND kidney without IRI (Figure 6A,B). In contrast, DATS+IRI kidneys normalized LCN2 levels comparable to ND kidneys and reduced the expression of TNF-α, IL-17, and IL-β by 0.6-fold, 0.5-fold, and 0.68-fold, respectively, from ND+IRI kidney levels (Figure 6A,B). The expression of inflammatory markers did not differ between ND and DATS kidneys (Figure 6A,B).

Recent studies suggest TGF-β signaling in endothelial cells contributes to the loss of peritubular capillaries in AKI and subsequent transition to chronic kidney disease (CKD) [49]. To determine whether TGF-β and Col1A1 markers are affected in renal IRI of aged kidneys, we quantified their expression. The expression of TGF-β was upregulated in the ND+IRI kidney by 1.9-fold and Col1A1 expression was increased by 4.4-fold compared to the ND kidney. In the DATS+IRI kidney, TGF-β and Col1A1 expression were downregulated significantly and were comparable to ND kidney (Figure 6C,D). Additionally, in the ND+IRI kidney, NF-kB expression was upregulated compared to the ND kidney without IRI and mitigated with DATS treatment (Figure 6C,D).

### 3.5. DATS Rescued Renal Blood Flow and Reduced Renovascular Resistance to the IRI Kidney

**Figure 7:**
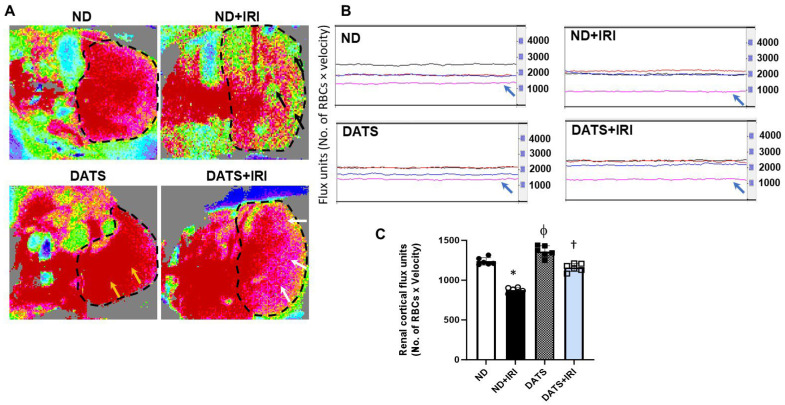
DATS improved renal cortical blood flow in the IRI kidney. (**A**) Representative laser Doppler flowmetry images showing renal cortical perfusion. The kidney is outlined by black dashed lines. Reduction of blood flow in ND+IRI kidney is shown as a low-intensity signal (green areas, black arrows), and improvement in blood flow is shown as pink areas (white arrows) in DATS+IRI kidney. DATS alone increased blood flow in the DATS kidney (yellow arrows). (**B**) Line traces from the kidney. Black trace is from the aorta, red trace is from the renal artery, blue trace is from the renal vein, and pink trace is from the renal cortex (blue arrow). (**C**) Data are expressed as mean ± SD, n = 6/group, *, ^ϕ^ *p* < 0.01 vs. ND, ^†^ *p* < 0.01 vs. ND+IRI.

Aging is associated with structural and functional changes in renal vasculature. To determine changes in the renal perfusion in the aged kidney following IRI, we measured cortical blood flow using laser Doppler flowmetry (MoorFLPI, Wilmington, DE, USA) at room temperature. In the ND+IRI kidney, cortical blood flow decreased significantly (29%) compared to the ND kidney that was not subjected to IRI (Figure 7A–C). DATS treatment alone increased blood flow by 9.9% compared to the ND kidney, and in the DATS+IRI kidney, cortical blood flow improved by 22.7% from the levels in ND+IRI (Figure 7A–C).

**Figure 8:**
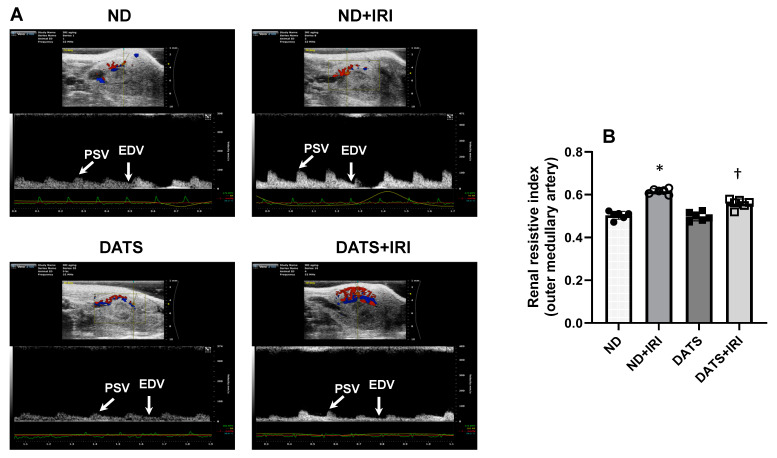
DATS treatment reduced the increased resistive index (RI) of outer medullary arteries caused by IRI in the aged kidney. (**A**) Representative ultrasound images of the kidney showing color Doppler of vessels in the outer cortico-medullary area. Pulsed-wave Doppler was placed on the outer medullary arteries to acquire data. The resistive index (RI) was measured using the formula RI = (PSV − EDV)/PSV. RI—resistive index, PSV—peak systolic velocity, EDV—end-diastolic velocity. (**B**) Values are mean ± SD, n = 6/group, * *p* < 0.01 vs. ND, ^†^ *p* < 0.01 vs. ND+IRI.

As the outer medullary region of the kidney is vulnerable to prolonged changes to blood flow following IRI, we measured the Doppler resistive index (RI) in the outer medullary artery by ultrasound to quantify blood flow alterations. In the ND+IRI kidney, the RI was increased significantly compared to the ND kidney (0.61 ± 0.01 vs. 0.50 ± 0.01) (Figure 8A,B). In the DATS kidney, RI was similar to ND. However, in the DATS+IRI kidney, RI decreased compared to ND+IRI (0.55 ± 0.02 vs. 0.61 ± 0.01) (Figure 8A,B).

### 3.6. DATS Ameliorated Renal Microvasculature and Function in the IRI Kidney

**Figure 9:**
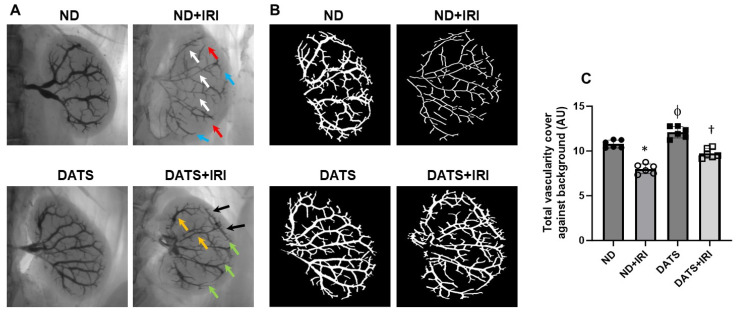
Renal IRI caused functional vascular rarefaction in the aged kidney and DATS treatment improved renal vascular density. (**A**) Representative barium sulfate angiography images of renal vasculature characterized by narrowing of lobar arteries (white arrows), loss of arcuate and interlobular branches (red and blue arrows, respectively), DATS rescues renal vasculature in WT+DATS+IRI kidney (yellow, green, and black arrows). (**B**) Vessel segmentation analysis images of total vasculature against the background. Vessel segmentation was conducted using VesSeg software (VesSeg_V0.1.4, University of Lubeck, Lubeck, Germany), (**C**) Data are mean ± SD, n = 6/group, *, ^ϕ^ *p* < 0.01 vs. ND, ^†^ *p* < 0.01 vs. ND+IRI.

To quantify changes in renal microvasculature, we used barium sulfate angiography. In the ND+IRI kidney, total vascular density was reduced compared to the ND kidney without IRI (10.7% ± 0.43 vs. 8.01% ± 0.54). The interlobar arteries showed narrowing, and there was a loss of arcuate and interlobular arteries in the cortex (Figure 9A–C). Rarefaction was predominant in the outer medullary region (Figure 9A,B). In the DATS kidney without IRI, total vascularity was increased compared to the ND kidney without IRI (12.1% ± 0.64 vs. 10.7% ± 0.43), and the interlobar arteries were dilated and interlobular arteries were prominent in the cortex (Figure 9A–C). In the DATS+IRI kidney, total vascularity increased (9.73% ± 0.54) compared to the ND+IRI kidney, as well as the interlobular artery diameter increased, and the medullary regions showed increased vasculature (Figure 9A–C).

**Figure 10:**
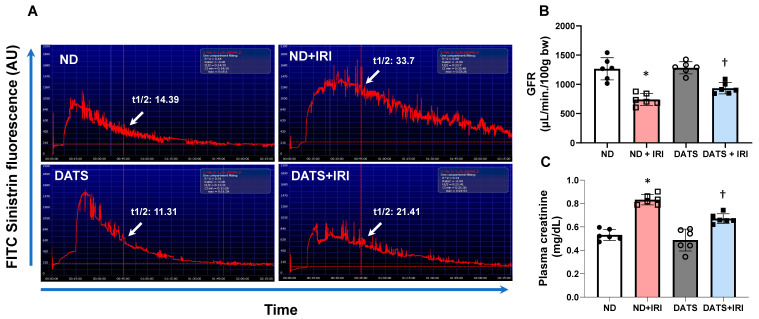
DATS rescued the aged kidney from severe dysfunction caused by IRI. (**A**) Representative images of glomerular filtration rate (GFR) estimated by non-invasive FITC-sinistrin method. (**B**) GFR was calculated using the formula, GFR [μL/min/100 g b.w.] = 14,616.8 [μL/100 g b.w.]/t_1/2_ (FITC-sinistrin) [min]. (**C**) Plasma creatinine was measured using QuantiChrom^TM^ creatinine assay kit by colorimetric method. Plasma creatinine concentration was calculated using the following formula: [(OD sample 5 − ODsample 0)/(OD standard 5 − OD standard 0)] × [STD] (mg/dL). Data are mean ± SD, n = 6/group, * *p* < 0.01 vs. ND, ^†^ *p* < 0.01 vs. ND+IRI.

Next, we measured the glomerular filtration rate (GFR) as an indicator of renal function. In the ND+IRI kidney, GFR decreased significantly (58.49%) compared to the ND kidney (Figure 10A,B). In the DATS kidney, GFR remained unchanged compared to the ND kidney (Figure 10A,B). In the DATS+IRI kidney, GFR improved by 15.21% from the GFR in the ND+IRI group (Figure 10A,B).

Plasma creatinine was measured to confirm renal dysfunction. In the ND+IRI group, plasma creatine was increased and correlated with declining GFR (Figure 10B,C). In the DATS+IRI group, creatinine levels decreased compared to the ND+IRI group (Figure 10C). There was no change in creatinine levels between the ND and DATS groups (Figure 10C).

## 4. Discussion

The production of endogenous hydrogen sulfide (H_2_S) is well-known for its cardioprotective functions, particularly in regulating vascular tone and promoting angiogenesis [50]. Additionally, H_2_S-induced S-sulfhydration plays a significant role in regulating angiogenesis and oxidative stress [51]. Furthermore, H_2_S has been shown to alleviate hyperhomocysteinemia-induced endothelial endoplasmic reticulum (ER) stress by sulfhydrating protein disulfide isomerase, thus reducing the progression of atherosclerosis [52]. Exogenous H_2_S has also been observed to improve outcomes in non-alcoholic fatty liver disease by inhibiting the ER stress/NLRP3 inflammasome pathway [53]. Moreover, targeted delivery of cystathionine-γ-lyase (CSE) plasmids to the infarcted myocardium has been found to reduce infarct size, improve cardiac function, and decrease oxidative stress, while also reducing ER stress [54]. This suggests that localized delivery of CSE, an enzyme that produces H_2_S, has cardioprotective effects by reducing stress [54]. However, it remains largely unclear whether diallyl trisulfide (DATS), an H_2_S generator, can modulate ER stress and angiogenesis in ischemia-reperfusion injury (IRI) in aging kidneys, particularly in females.

The recovery of renal microvascular function is crucial to minimizing IRI-induced damage and progression to CKD [55]. AKI and progression of CKD are linked to activation of ER stress pathways [56,57,58] and gender differences are reported in ER response to stress in AKI in young mice models [59]. However, there is a paucity of literature regarding ER stress in aged kidney following IRI. Therefore, in the present study, we determined the effects of age-related response to ER stress during renal IRI and whether DATS, a hydrogen sulfide (H_2_S) donor, can mitigate AKI pathology in female kidneys via the regulation of ER stress. Diallyl trisulfide (DATS) is a garlic-derived organosulfide that releases H_2_S from red blood cells upon reaction with thiols such as cysteine or glutathione (GSH). A proton shuffle from the thiol to allyl perthiol anion forms allyl perthiol by thiol/disulfide exchange, thus releasing H_2_S [60,61]. Our results indicated that following IRI, H_2_S production is decreased in aged female kidney, and associated with accumulation of excess ER stress proteins, downregulation of angiogenesis markers, and PI3K, AKT, p-mTOR, and pMAPK signaling pathways. The changes were accompanied by upregulation of inflammatory cytokines and fibrosis markers. Further, the reduction of renal vascular density and increased resistive index (RI) reduced renal blood flow, worsening renal function in IRI female kidney vs. control. DATS treatment suppressed ER stress, upregulated angiogenesis markers to increase vascularity, and restored blood flow to improve function and attenuated inflammation and fibrosis. Taken together, our results suggest that DATS protects the aged female kidney by modulating ER stress response and enhancing angiogenesis.

Oxygen deprivation during renal ischemia drives adaptive processes that include unfolded protein response (UPR) [4]. Previous studies demonstrated that ER stress regulates the production of angiogenic factors, VEGF-A and bFGF in renal IRI and diabetic retinopathy promoting angiogenesis [62,63]. In other studies, ER stress was shown to induce anti-angiogenesis signaling via IRE1α and PERK pathway in the retina, CHOP in hind-limb ischemia, and CREB3L1 in mammary tumor model [64,65,66]. The above studies suggest that while ER stress and angiogenesis are linked, a balance between protein folding and protein degradation determines whether the pro-survival or pro-death response predominates to induce angiogenesis and reduction of damage during renal IRI. In the present study, the upregulation of HIF1α, VEGF-A, and EPO in the presence of increased p-IRE1α, p-PERK, and ATF6 suggests an interaction between hypoxia, ER response, and angiogenesis mechanisms during renal IRI (Figure 1, Figure 2 and Figure 3). However, eNOS expression was decreased in the IRI kidneys suggesting impaired endothelial function (Figure 3). CHOP is a transcriptional factor that is induced by IRE1α, PERK, and ATF6 [67], and in a previous study, Loinard et al. demonstrated that CHOP represses eNOS [65]. Our findings show that reduced eNOS in IRI kidney is therefore secondary to increased CHOP expression (Figure 1D and Figure 3). H_2_S has been shown to play a protective role in several disease states by suppressing ER stress [68]. Further, it is known to promote angiogenesis by inducing VEGF [69,70]. The results from the present study suggest that H_2_S reduced ER stress and increased eNOS expression and blood flow in the IRI kidney (Figure 3 and Figure 7).

In mammalian cells, H_2_S is produced by the enzymes of the transsulfuration pathway, cystathionine β-synthase (CBS), cystathionine γ-lyase (CSE), and the mitochondrial enzyme, 3-mercaptopyruvate sulfurtransferase (3-MST) that is coupled with cysteine aminotransferase (CAT) [71]. Exogenous H_2_S supplementation has been shown to act as an antioxidant, anti-inflammatory, and anti-apoptotic molecule in several pathological conditions [72,73]. In myocardial IRI, H_2_S offers cardioprotection by persulfidation of the K_ATP_ channel leading to vasorelaxation [74]. Previously, in IRI of aging kidney, we and others reported a decreased H_2_S content in the plasma and kidney, and exogenous supplementation of H_2_S mitigates renal IRI in male rodents [26,75] and a miniature young female swine model [76]. Sexual dimorphism has been reported for H_2_S production, sensitivity to vascular tissue, and sulfide bioavailability in animal models and patients with cardiovascular disease [77,78]. However, to our knowledge, there are no data from aging studies reporting changes in H_2_S levels in the aged female kidneys and the response to IRI. It is important to note that while females are resistant to IRI at a younger age [79], their susceptibility to IRI increases with aging [80]. We found that the H_2_S levels in the kidney following IRI were lower in females in the present study (Figure 1A) compared to males previously [26] and the control in this study. There was no difference in H_2_S levels between genders following H_2_S supplementation in the IRI group, suggesting that H_2_S level recovery is similar in aged kidneys in females and males.

Inositol-requiring enzyme-1 (IRE1) is an endoplasmic reticulum-bound kinase/endoribonuclease (RNase) that regulates unfolded protein response (UPR) [81]. IRE1 on the ER membrane senses misfolded proteins leading to IRE1 oligomerization and activation of its kinase, RNase. Under pathophysiological conditions, the fine balance between protein load and protein folding capacity is disrupted leading to the activation of three ER transmembrane sensors, i.e., inositol requiring enzyme 1 (IRE1), protein kinase RNA-like ER eukaryotic initiation factor-2α kinase (PERK), and activating transcription factor 6 (ATF6) [7]. Upon activation the proteins initiate signaling and a transcriptional network termed UPR, which participates in upregulating inflammatory process [82], an indication of ER stress. In the present study, involving aged female kidney, all ER proteins were upregulated indicating ER stress (Figure 1B–C and Figure 2A,B). The recent literature suggests that H_2_S treatment protects the heart during diabetic cardiomyopathy and ischemic injury by suppressing ER stress [27,83]. Our data agree with the earlier studies and confirm a similar protective mechanism in aged kidney IRI. The prolongation of ER stress leads to apoptosis via activation of transcription factor 4-C/EBP homology protein (ATF4-CHOP)-induction in IRI kidney [84]. ER function is dependent on a multifunctional integral membrane, luminal chaperones, folding enzymes, and sensor molecules such as heat shock proteins, GRP78 and GRP94 (glucose-related protein 78/94). Under stress, GRP78 and GRP94 chaperones are upregulated to enhance protein folding by ER in CKD [11]. Our findings indicate that in IRI of aged female kidneys, mRNA of these stress genes is upregulated, and DATS mitigated their expression (Figure 1D). Taken together, our results suggest that DATS modulates key ER stress genes that include IRE1α, PERK, CHOP, ATF4, ATF6, GRP78, and GRP94 in IRI-induced AKI in aging female mice.

Hypoxia-inducible factors (HIFs) are the master transcription factors responsible for gene regulation in hypoxia/ischemia [85] by inducing the expression of adaptive gene products, such as vascular endothelial growth factor a (VEGF-A), erythropoietin (EPO), and endothelial nitric oxide synthase (eNOS) [86,87]. In a glycerol-induced AKI rodent model, it was shown that the HIF-1α and VEGF-A were increased in young male rats [88]. Erythropoietin (EPO) is a cytokine that is produced by the kidney and induced under hypoxic conditions [89]. In a bilateral renal IRI isogenic mice model, there was a five-fold increase in EPO that contributed to the abrogation of kidney IRI [90]. IR decreases renal eNOS expression in male adult rat model [91]. VEGF and EPO promote angiogenesis and eNOS regulates vascular tone to optimize blood flow to organs. Our results indicate that IRI increases angiogenesis markers but eNOS expression is diminished. DATS further enhanced the expression of relevant angiogenesis markers and increased eNOS (Figure 3A,B), indicating angiogenesis (Figure 4A,B) and improved endothelial function in the aging female IRI kidney. However, whether age-matched IRI-induced adaptive protein response and angiogenesis is similar in male kidney is yet to be investigated.

Angiogenesis is the physiological process of forming new blood vessels or repairing pre-existing blood vessels during growth or following injury [92]. VEGF-A is a member of the VEGF family that plays a key role in angiogenesis, and several signaling cascades are vital for this process to happen. For example, growth factors initiate PI3K/AKT/mTOR cascade as well as MAPK pathways for endothelial survival, vascular permeability, and migratory or proliferative phenotypes [93]. It was shown that PI3K/AKT activation attenuated AKI in adult male Sprague–Dawley rats [94]. In addition, AKI-induced oxidative stress was regulated by the AKT/mTOR pathway in young male mice [95]. Our data indicates that PI3K/AKT/mTOR and MAPK kinase pathways are inhibited in the aging female kidney following IRI and DATS treatment normalizes their activity (Figure 5A,B). To our knowledge, this is the first report that indicates that angiogenic signaling cascades are downregulated in aging female AKI and that DATS treatment restores the expression and activity.

Interestingly, the PAM (PI3K/AKT/mTOR) signaling pathway has been reported to promote cell survival, cell growth, and cell cycle progression [96]. This pathway is highly regulated by multiple interactions with several other signaling pathways, especially in the context of cancer development [96]. Our results indicated that DATS, a source of H_2_S, is protective against renal IRI in aging through normalizing PAM signaling pathway. Conversely, an in vitro study demonstrated that the topical application of NaHS, which serves as a direct source of H_2_S, inhibited the PAM signaling pathway in human hepatocellular carcinoma (HCC) cells [97]. The differences in outcomes may be attributed to the distinct forms of H_2_S being used. While NaHS provides a direct source, DATS requires metabolism by glutathione in red blood cells, leading to a slower release of H_2_S [98]. Other factors could also contribute to these differing outcomes, as the experimental conditions vary. These include the comparison of in vitro cellular responses versus in vivo H_2_S responses, cancerous cells versus ischemic tissue, and liver cells versus renal tissue.

It is also important to note that oxidative stress is a common pathway and parameter for IRI, as reported by many investigators [99,100]. In this study, our goal was to investigate IRI-induced endoplasmic reticulum (ER) stress and whether DATS, an H_2_S generator, could mitigate this stress. Our findings indicate that DATS does indeed provide such mitigation. Additionally, DATS has been reported to have anticancer activity, which is attributed to the induction of apoptosis that occurs in a cell cycle-dependent manner, specifically by transitioning from the G2/M phase to the G1 phase [101,102]. We believe that the discrepancy between DATS’s protective effects against IRI and its induction of cellular apoptosis can be explained by the difference between in vitro cancerous cells and in vivo IRI pathology in aging kidneys. Nonetheless, further and larger in vivo studies are needed to clarify these apparent differences in outcomes.

Lipocalin-2 (LCN-2) is a biomarker of kidney injury [103] and its levels are reported to increase, both in serum and urine, in patients with AKI [104], whereas tumor necrosis factor-alpha (TNF-α) is a cytokine and major regulator of inflammatory response following kidney injury [105]. Further, the upregulation of inflammatory molecules, such as IL-17 and IL-β1 is linked to AKI [106,107]. Our results support the earlier findings and show that DATS treatment normalizes their expression in the kidney indicating that DATS mitigates kidney injury and inflammation via release of H_2_S (Figure 6A,B). TGF-β causes acute tubular injury and inflammation and has a mechanistic link between acute injury and progression to CKD [108]. TGF-β is a potent stimulator of collagen formation [49,109] that in concert with nuclear factor-κB (NF-κB) activation promotes the production of proinflammatory proteins and stimulation of renal fibrosis following AKI [110]. We show that DATS suppresses the expression of TGF-β, Col1A1, and NF-κB to alleviate renal inflammation and fibrosis (Figure 6C,D).

AKI leads to impaired renal blood flow which is a contributory factor to diminished GFR [12]. Renal RI is a reliable diagnostic tool that is used in intensive care units to predict and assess AKI severity [111]. We have previously shown that renal vascularity is diminished in young and aged male mice, but aged mice exhibited less vascularity compared to young mice with AKI [26]. Further, we demonstrated that GYY4137, an H_2_S-releasing compound, improved vascular density in AKI mice. Here, we show that DATS increases renal blood flow by lowering the vascular resistance in the microvessels, thus improving renal function by increasing GFR and lowering plasma creatinine in the female kidney following IRI (Figure 7, Figure 8, Figure 9 and Figure 10).

It is noteworthy to mention that DATS exhibits structural similarities to endogenous metabolites generated during anaerobic cysteine metabolism, which leads to the formation of sulfane sulfur-containing compounds and H_2_S [112]. Both sulfane sulfur and H_2_S are classified as reactive sulfur species (RSS) produced under physiological conditions. Research suggests that the signaling molecule responsible for the biological effects of RSS, including the S-sulfhydration of proteins, is sulfane sulfur rather than H_2_S [112,113]. While it is possible that a similar mechanism could be at play in our study, it is theoretically unlikely. This is primarily because our method of DATS administration was through dietary intake, whereas previous studies used intraperitoneal injections and topical in vitro applications [112,113]. Furthermore, in our research, the IRI in the kidneys did not deliver completely anaerobic environment for the duration of the experiments, and the DATS regimen was administered for three weeks prior to IRI induction and continued for one week afterward. Nonetheless, whether the benefits of DATS arise from RSS production or are solely due to H_2_S generation requires further investigation.

## 5. Conclusions

We demonstrate that in aged female mice, renal IRI impairs function significantly, which is similar to aged male mice with renal IRI. In both genders, impaired function is in part a consequence of H_2_S deficiency. In the aged female kidney IRI, ER stress is increased and associated with diminished angiogenesis involving PI3K, AKT, mTOR, and MAPK signaling pathways. Inflammation and fibrosis are additional components of AKI. A decrease in vascularity and eNOS reduces blood flow and supposedly endothelial function. Increasing H_2_S levels and bioavailability by DATS modulates ER stress, angiogenesis, and targets inflammatory and fibrosis molecules to mitigate AKI in the aged female kidney. Gender differences in ER stress response to aged male kidney IRI require further investigation.

## Figures and Tables

**Table 1:** Primer sequences.

Gene	Forward (5′–3′)	Reverse (5′–3′)
*GRP78*	TTCAGCCAATTATCAGCAAACTCT	TTTTCTGATGTATCCTCTTCACCAGT
*GRP94*	AAGAATGAAGGAAAAACAGGACAAAA	CAAATGGAGAAGATTCCGCC
*ATF4*	GGGTTCTGTCTTCCACTCCA	AAGCAGCAGAGTCAGGCTTTC
*CHOP*	CCACCACACCTGAAAGCAGAA	AGGTGAAAGGCAGGGACTCA

## Data Availability

The original contributions presented in the study are included in the article; further inquiries can be directed to the corresponding author.

## Abbreviations

3-MST: 3-mercaptopyruvate sulfurtransferase; AKI, acute kidney injury; AKT, protein kinase B (a serine/threonine kinase); ATF6, activating transcription factor 6; CAT, cysteine aminotransferase; CBS, cystathionine β-synthase; CSE, cystathionine γ-lyase; DATS, diallyl trisulfide; eNOS, endothelial nitric oxide synthase; EPO, erythropoietin; ER, endoplasmic reticulum; GFR, glomerular filtration rate; H_2_S, hydrogen sulfide; HIF, hypoxia-inducible factor; IL, interleukin; IRE1, inositol requiring protein 1; IRI, ischemia-reperfusion injury; LCN2, lipocalin-2, also known as neutrophil gelatinase-associated lipocalin (NGAL); MAPK, mitogen-activated protein kinase; mTOR, mammalian target of rapamycin; ND, normal diet; NF-kB, nuclear factor kappa-B; p-, phosphorylated; PERK, protein kinase RNA-like ER eukaryotic initiation factor-2α kinase; PI3K, phosphoinositide 3-kinase; TGF, transforming growth factor; TNF, tumor necrosis factor; UPR, unfolded protein response; VEGF-A, vascular endothelial growth factor-A; WT, wild-type.

## References

[1] Nieuwenhuijs-Moeke G.J., Pischke S.E., Berger S.P., Sanders J.S.F., Pol R.A., Struys M., Ploeg R.J., Leuvenink H.G.D. (2020). Ischemia and Reperfusion Injury in Kidney Transplantation: Relevant Mechanisms in Injury and Repair. J. Clin. Med..

[2] Tammaro A., Kers J., Scantlebery A.M.L., Florquin S. (2020). Metabolic Flexibility and Innate Immunity in Renal Ischemia Reperfusion Injury: The Fine Balance Between Adaptive Repair and Tissue Degeneration. Front. Immunol..

[3] Thadhani R., Pascual M., Bonventre J.V. (1996). Acute renal failure. N. Engl. J. Med..

[4] Yan M., Shu S., Guo C., Tang C., Dong Z. (2018). Endoplasmic reticulum stress in ischemic and nephrotoxic acute kidney injury. Ann. Med..

[5] Taniguchi M., Yoshida H. (2015). Endoplasmic reticulum stress in kidney function and disease. Curr. Opin. Nephrol. Hypertens..

[6] Noh M.R., Kim J.I., Han S.J., Lee T.J., Park K.M. (2015). C/EBP homologous protein (CHOP) gene deficiency attenuates renal ischemia/reperfusion injury in mice. Biochim. Biophys. Acta.

[7] Battson M.L., Lee D.M., Gentile C.L. (2017). Endoplasmic reticulum stress and the development of endothelial dysfunction. Am. J. Physiol. Heart Circ. Physiol..

[8] Tam A.B., Mercado E.L., Hoffmann A., Niwa M. (2012). ER stress activates NF-kappaB by integrating functions of basal IKK activity, IRE1 and PERK. PLoS ONE.

[9] Shu S., Zhu J., Liu Z., Tang C., Cai J., Dong Z. (2018). Endoplasmic reticulum stress is activated in post-ischemic kidneys to promote chronic kidney disease. Ebiomedicine.

[10] Porter A.W., Brodsky J.L., Buck T.M. (2022). Emerging links between endoplasmic reticulum stress responses and acute kidney injury. Am. J. Physiol. Cell Physiol..

[11] Wu D., Huang L.F., Chen X.C., Huang X.R., Li H.Y., An N., Tang J.X., Liu H.F., Yang C. (2023). Research progress on endoplasmic reticulum homeostasis in kidney diseases. Cell Death Dis..

[12] Basile D.P., Anderson M.D., Sutton T.A. (2012). Pathophysiology of acute kidney injury. Compr. Physiol..

[13] Horbelt M., Lee S.Y., Mang H.E., Knipe N.L., Sado Y., Kribben A., Sutton T.A. (2007). Acute and chronic microvascular alterations in a mouse model of ischemic acute kidney injury. Am. J. Physiol. Ren. Physiol..

[14] Basile D.P., Fredrich K., Chelladurai B., Leonard E.C., Parrish A.R. (2008). Renal ischemia reperfusion inhibits VEGF expression and induces ADAMTS-1, a novel VEGF inhibitor. Am. J. Physiol. Ren. Physiol..

[15] Kwon O., Hong S.M., Sutton T.A., Temm C.J. (2008). Preservation of peritubular capillary endothelial integrity and increasing pericytes may be critical to recovery from postischemic acute kidney injury. Am. J. Physiol. Ren. Physiol..

[16] Kwiatkowska E., Kwiatkowski S., Dziedziejko V., Tomasiewicz I., Domanski L. (2023). Renal Microcirculation Injury as the Main Cause of Ischemic Acute Kidney Injury Development. Biology.

[17] Bonventre J.V., Yang L. (2011). Cellular pathophysiology of ischemic acute kidney injury. J. Clin. Investig..

[18] Basile D.P. (2007). The endothelial cell in ischemic acute kidney injury: Implications for acute and chronic function. Kidney Int..

[19] Maringer K., Sims-Lucas S. (2016). The multifaceted role of the renal microvasculature during acute kidney injury. Pediatr. Nephrol..

[20] Dimke H., Sparks M.A., Thomson B.R., Frische S., Coffman T.M., Quaggin S.E. (2015). Tubulovascular cross-talk by vascular endothelial growth factor a maintains peritubular microvasculature in kidney. J. Am. Soc. Nephrol..

[21] Dong Y., Fernandes C., Liu Y., Wu Y., Wu H., Brophy M.L., Deng L., Song K., Wen A., Wong S. (2017). Role of endoplasmic reticulum stress signalling in diabetic endothelial dysfunction and atherosclerosis. Diabetes Vasc. Dis. Res..

[22] Kassan M., Galan M., Partyka M., Saifudeen Z., Henrion D., Trebak M., Matrougui K. (2012). Endoplasmic reticulum stress is involved in cardiac damage and vascular endothelial dysfunction in hypertensive mice. Arterioscler. Thromb. Vasc. Biol..

[23] Zhang C., Cai Y., Adachi M.T., Oshiro S., Aso T., Kaufman R.J., Kitajima S. (2001). Homocysteine induces programmed cell death in human vascular endothelial cells through activation of the unfolded protein response. J. Biol. Chem..

[24] Sanson M., Auge N., Vindis C., Muller C., Bando Y., Thiers J.C., Marachet M.A., Zarkovic K., Sawa Y., Salvayre R. (2009). Oxidized low-density lipoproteins trigger endoplasmic reticulum stress in vascular cells: Prevention by oxygen-regulated protein 150 expression. Circ. Res..

[25] Lenna S., Han R., Trojanowska M. (2014). Endoplasmic reticulum stress and endothelial dysfunction. IUBMB Life.

[26] Pushpakumar S., Kundu S., Weber G., Sen U. (2021). Exogenous hydrogen sulfide and miR-21 antagonism attenuates macrophage-mediated inflammation in ischemia reperfusion injury of the aged kidney. Geroscience.

[27] Li F., Luo J., Wu Z., Xiao T., Zeng O., Li L., Li Y., Yang J. (2016). Hydrogen sulfide exhibits cardioprotective effects by decreasing endoplasmic reticulum stress in a diabetic cardiomyopathy rat model. Mol. Med. Rep..

[28] Li C., Hu M., Wang Y., Lu H., Deng J., Yan X. (2015). Hydrogen sulfide preconditioning protects against myocardial ischemia/reperfusion injury in rats through inhibition of endo/sarcoplasmic reticulum stress. Int. J. Clin. Exp. Pathol..

[29] Zhong H., Yu H., Chen J., Sun J., Guo L., Huang P., Zhong Y. (2020). Hydrogen Sulfide and Endoplasmic Reticulum Stress: A Potential Therapeutic Target for Central Nervous System Degeneration Diseases. Front. Pharmacol..

[30] Zou W., Yuan J., Tang Z.J., Wei H.J., Zhu W.W., Zhang P., Gu H.F., Wang C.Y., Tang X.Q. (2017). Hydrogen sulfide ameliorates cognitive dysfunction in streptozotocin-induced diabetic rats: Involving suppression in hippocampal endoplasmic reticulum stress. Oncotarget.

[31] Yang R., Teng X., Li H., Xue H.M., Guo Q., Xiao L., Wu Y.M. (2016). Hydrogen Sulfide Improves Vascular Calcification in Rats by Inhibiting Endoplasmic Reticulum Stress. Oxid. Med. Cell. Longev..

[32] Wang M., Wang Z., Miao Y., Wei H., Peng J., Zhou Y. (2022). Diallyl Trisulfide Promotes Placental Angiogenesis by Regulating Lipid Metabolism and Alleviating Inflammatory Responses in Obese Pregnant Mice. Nutrients.

[33] Puccinelli M.T., Stan S.D. (2017). Dietary Bioactive Diallyl Trisulfide in Cancer Prevention and Treatment. Int. J. Mol. Sci..

[34] Disbrow E., Stokes K.Y., Ledbetter C., Patterson J., Kelley R., Pardue S., Reekes T., Larmeu L., Batra V., Yuan S. (2021). Plasma hydrogen sulfide: A biomarker of Alzheimer’s disease and related dementias. Alzheimers Dement..

[35] Guzel C., Yesiltas S., Daskaya H., Uysal H., Sumer I., Turkay M. (2019). The effect of gender on acute kidney injury developing in the intensive care unit. Hippokratia.

[36] Sawhney S., Bell S., Black C., Christiansen C.F., Heide-Jorgensen U., Jensen S.K., Ronksley P.E., Tan Z., Tonelli M., Walker H. (2022). Harmonization of epidemiology of acute kidney injury and acute kidney disease produces comparable findings across four geographic populations. Kidney Int..

[37] Kim J., Cannon B.A., Freeman L.E., Tan S., Knych H.K., Kendall L.V. (2023). High-dose Meloxicam Provides Improved Analgesia in Female CD1 Mice: A Pharmacokinetic and Efficacy Study. J. Am. Assoc. Lab. Anim. Sci..

[38] Kendall L.V., Bailey A.L., Singh B., McGee W. (2022). Toxic Effects of High-dose Meloxicam and Carprofen on Female CD1 Mice. J. Am. Assoc. Lab. Anim. Sci..

[39] Kundu S., Pushpakumar S., Sen U. (2015). MMP-9- and NMDA receptor-mediated mechanism of diabetic renovascular remodeling and kidney dysfunction: Hydrogen sulfide is a key modulator. Nitric Oxide Biol. Chem. Off. J. Nitric Oxide Soc..

[40] Pushpakumar S., Ren L., Juin S.K., Majumder S., Kulkarni R., Sen U. (2020). Methylation-dependent antioxidant-redox imbalance regulates hypertensive kidney injury in aging. Redox Biol..

[41] Pushpakumar S., Kundu S., Pryor T., Givvimani S., Lederer E., Tyagi S.C., Sen U. (2013). Angiotensin-II induced hypertension and renovascular remodelling in tissue inhibitor of metalloproteinase 2 knockout mice. J. Hypertens..

[42] Pushpakumar S., Ren L., Kundu S., Gamon A., Tyagi S.C., Sen U. (2017). Toll-like Receptor 4 Deficiency Reduces Oxidative Stress and Macrophage Mediated Inflammation in Hypertensive Kidney. Sci. Rep..

[43] Schock-Kusch D., Sadick M., Henninger N., Kraenzlin B., Claus G., Kloetzer H.M., Weiss C., Pill J., Gretz N. (2009). Transcutaneous measurement of glomerular filtration rate using FITC-sinistrin in rats. Nephrol. Dial. Transplant..

[44] Schrijvers B.F., Flyvbjerg A., De Vriese A.S. (2004). The role of vascular endothelial growth factor (VEGF) in renal pathophysiology. Kidney Int..

[45] Karar J., Maity A. (2011). PI3K/AKT/mTOR Pathway in Angiogenesis. Front. Mol. Neurosci..

[46] Song Y.Y., Liang D., Liu D.K., Lin L., Zhang L., Yang W.Q. (2023). The role of the ERK signaling pathway in promoting angiogenesis for treating ischemic diseases. Front. Cell Dev. Biol..

[47] Hosszu A., Fekete A., Szabo A.J. (2020). Sex differences in renal ischemia-reperfusion injury. Am. J. Physiol. Ren. Physiol..

[48] Kang K.P., Lee J.E., Lee A.S., Jung Y.J., Kim D., Lee S., Hwang H.P., Kim W., Park S.K. (2014). Effect of gender differences on the regulation of renal ischemia-reperfusion-induced inflammation in mice. Mol. Med. Rep..

[49] Gewin L.S. (2019). Transforming Growth Factor-beta in the Acute Kidney Injury to Chronic Kidney Disease Transition. Nephron.

[50] Huang S., Li H., Ge J. (2015). A cardioprotective insight of the cystathionine gamma-lyase/hydrogen sulfide pathway. Int. J. Cardiol. Heart Vasc..

[51] Gupta R., Sahu M., Tripathi R., Ambasta R.K., Kumar P. (2022). Protein S-sulfhydration: Unraveling the prospective of hydrogen sulfide in the brain, vasculature and neurological manifestations. Ageing Res. Rev..

[52] Jiang S., Xu W., Chen Z., Cui C., Fan X., Cai J., Gong Y., Geng B. (2021). Hydrogen sulphide reduces hyperhomocysteinaemia-induced endothelial ER stress by sulfhydrating protein disulphide isomerase to attenuate atherosclerosis. J. Cell Mol. Med..

[53] Fu X., Zhang Q., Chen Y., Li Y., Wang H. (2025). Exogenous hydrogen sulfide improves non-alcoholic fatty liver disease by inhibiting endoplasmic reticulum stress/NLRP3 inflammasome pathway. Mol. Cell Biochem..

[54] Wang Q., Xue X., Wang P., Yu Y., Wang J., Jiang Q., Xiao J. (2024). Angiotensin 1 peptide-conjugated CdSe/ZnS quantum dots for cardiac-specific hydrogen sulfide targeted therapy in myocardial ischemia-reperfusion injury. Front. Pharmacol..

[55] Basile D.P., Yoder M.C. (2014). Renal endothelial dysfunction in acute kidney ischemia reperfusion injury. Cardiovasc. Hematol. Disord. Drug Targets.

[56] Li C., Krothapalli S., Chen Y.M. (2023). Targeting Endoplasmic Reticulum for Novel Therapeutics and Monitoring in Acute Kidney Injury. Nephron.

[57] Gallazzini M., Pallet N. (2018). Endoplasmic reticulum stress and kidney dysfunction. Biol. Cell.

[58] Ricciardi C.A., Gnudi L. (2020). The endoplasmic reticulum stress and the unfolded protein response in kidney disease: Implications for vascular growth factors. J. Cell. Mol. Med..

[59] Hodeify R., Megyesi J., Tarcsafalvi A., Mustafa H.I., Hti Lar Seng N.S., Price P.M. (2013). Gender differences control the susceptibility to ER stress-induced acute kidney injury. Am. J. Physiol. Ren. Physiol..

[60] Cai Y.R., Hu C.H. (2017). Computational Study of H(2)S Release in Reactions of Diallyl Polysulfides with Thiols. J. Phys. Chem. B.

[61] Liang D., Wu H., Wong M.W., Huang D. (2015). Diallyl Trisulfide Is a Fast H2S Donor, but Diallyl Disulfide Is a Slow One: The Reaction Pathways and Intermediates of Glutathione with Polysulfides. Org. Lett..

[62] Bouvier N., Fougeray S., Beaune P., Thervet E., Pallet N. (2012). The unfolded protein response regulates an angiogenic response by the kidney epithelium during ischemic stress. J. Biol. Chem..

[63] Zhong Y., Li J., Chen Y., Wang J.J., Ratan R., Zhang S.X. (2012). Activation of endoplasmic reticulum stress by hyperglycemia is essential for Muller cell-derived inflammatory cytokine production in diabetes. Diabetes.

[64] Binet F., Mawambo G., Sitaras N., Tetreault N., Lapalme E., Favret S., Cerani A., Leboeuf D., Tremblay S., Rezende F. (2013). Neuronal ER stress impedes myeloid-cell-induced vascular regeneration through IRE1alpha degradation of netrin-1. Cell Metab..

[65] Loinard C., Zouggari Y., Rueda P., Ramkhelawon B., Cochain C., Vilar J., Recalde A., Richart A., Charue D., Duriez M. (2012). C/EBP homologous protein-10 (CHOP-10) limits postnatal neovascularization through control of endothelial nitric oxide synthase gene expression. Circulation.

[66] Mellor P., Deibert L., Calvert B., Bonham K., Carlsen S.A., Anderson D.H. (2013). CREB3L1 is a metastasis suppressor that represses expression of genes regulating metastasis, invasion, and angiogenesis. Mol. Cell. Biol..

[67] Todd D.J., Lee A.H., Glimcher L.H. (2008). The endoplasmic reticulum stress response in immunity and autoimmunity. Nat. Rev. Immunol..

[68] Wang H., Shi X., Qiu M., Lv S., Liu H. (2020). Hydrogen Sulfide Plays an Important Protective Role through Influencing Endoplasmic Reticulum Stress in Diseases. Int. J. Biol. Sci..

[69] Szabo C., Papapetropoulos A. (2011). Hydrogen sulphide and angiogenesis: Mechanisms and applications. Br. J. Pharmacol..

[70] Zhang Y.X., Jing M.R., Cai C.B., Zhu S.G., Zhang C.J., Wang Q.M., Zhai Y.K., Ji X.Y., Wu D.D. (2023). Role of hydrogen sulphide in physiological and pathological angiogenesis. Cell Prolif..

[71] Kimura H. (2014). Production and physiological effects of hydrogen sulfide. Antioxid. Redox Signal..

[72] Filipovic M.R., Zivanovic J., Alvarez B., Banerjee R. (2018). Chemical Biology of H(2)S Signaling through Persulfidation. Chem. Rev..

[73] Feng J., Lu X., Li H., Wang S. (2022). The roles of hydrogen sulfide in renal physiology and disease states. Ren. Fail..

[74] Johansen D., Ytrehus K., Baxter G.F. (2006). Exogenous hydrogen sulfide (H2S) protects against regional myocardial ischemia-reperfusion injury--Evidence for a role of K ATP channels. Basic Res. Cardiol..

[75] Lobb I., Zhu J., Liu W., Haig A., Lan Z., Sener A. (2014). Hydrogen sulfide treatment ameliorates long-term renal dysfunction resulting from prolonged warm renal ischemia-reperfusion injury. Can. Urol. Assoc. J..

[76] Sekijima M., Sahara H., Miki K., Villani V., Ariyoshi Y., Iwanaga T., Tomita Y., Yamada K. (2017). Hydrogen sulfide prevents renal ischemia-reperfusion injury in CLAWN miniature swine. J. Surg. Res..

[77] Andrey M.n., Natalia Z., Alexandr O., Iryna P. (2014). P1 Gender dimorphism of hydrogen sulfide production and physiological effects in cardiovascular system. Nitric Oxide Biol. Chem. Off. J. Nitric Oxide Soc..

[78] Rajpal S., Katikaneni P., Deshotels M., Pardue S., Glawe J., Shen X., Akkus N., Modi K., Bhandari R., Dominic P. (2018). Total sulfane sulfur bioavailability reflects ethnic and gender disparities in cardiovascular disease. Redox Biol..

[79] Tanaka R., Tsutsui H., Ohkita M., Takaoka M., Yukimura T., Matsumura Y. (2013). Sex differences in ischemia/reperfusion-induced acute kidney injury are dependent on the renal sympathetic nervous system. Eur. J. Pharmacol..

[80] Privratsky J.R., Fuller M., Raghunathan K., Ohnuma T., Bartz R.R., Schroeder R., Price T.M., Martinez M.R., Sigurdsson M.I., Mathis M.R. (2023). Postoperative Acute Kidney Injury by Age and Sex: A Retrospective Cohort Association Study. Anesthesiology.

[81] Hamid S.M., Citir M., Terzi E.M., Cimen I., Yildirim Z., Dogan A.E., Kocaturk B., Onat U.I., Arditi M., Weber C. (2020). Inositol-requiring enzyme-1 regulates phosphoinositide signaling lipids and macrophage growth. EMBO Rep..

[82] Garg A.D., Kaczmarek A., Krysko O., Vandenabeele P., Krysko D.V., Agostinis P. (2012). ER stress-induced inflammation: Does it aid or impede disease progression?. Trends Mol. Med..

[83] Chen J., Gao J., Sun W., Li L., Wang Y., Bai S., Li X., Wang R., Wu L., Li H. (2016). Involvement of exogenous H2S in recovery of cardioprotection from ischemic post-conditioning via increase of autophagy in the aged hearts. Int. J. Cardiol..

[84] Rozpedek W., Pytel D., Mucha B., Leszczynska H., Diehl J.A., Majsterek I. (2016). The Role of the PERK/eIF2alpha/ATF4/CHOP Signaling Pathway in Tumor Progression During Endoplasmic Reticulum Stress. Curr. Mol. Med..

[85] Shu S., Wang Y., Zheng M., Liu Z., Cai J., Tang C., Dong Z. (2019). Hypoxia and Hypoxia-Inducible Factors in Kidney Injury and Repair. Cells.

[86] Lemus-Varela M.L., Flores-Soto M.E., Cervantes-Munguia R., Torres-Mendoza B.M., Gudino-Cabrera G., Chaparro-Huerta V., Ortuno-Sahagun D., Beas-Zarate C. (2010). Expression of HIF-1 alpha, VEGF and EPO in peripheral blood from patients with two cardiac abnormalities associated with hypoxia. Clin. Biochem..

[87] Rodriguez-Miguelez P., Lima-Cabello E., Martinez-Florez S., Almar M., Cuevas M.J., Gonzalez-Gallego J. (2015). Hypoxia-inducible factor-1 modulates the expression of vascular endothelial growth factor and endothelial nitric oxide synthase induced by eccentric exercise. J Appl Physiol (1985).

[88] Mao H., Jiang C., Xu L., Chen D., Liu H., Xu Y., Ma K., Wang M. (2020). Ginsenoside protects against AKI via activation of HIF-1alpha and VEGF-A in the kidney-brain axis. Int. J. Mol. Med..

[89] Rey F., Balsari A., Giallongo T., Ottolenghi S., Di Giulio A.M., Samaja M., Carelli S. (2019). Erythropoietin as a Neuroprotective Molecule: An Overview of Its Therapeutic Potential in Neurodegenerative Diseases. ASN Neuro.

[90] Correa-Costa M., Gallo D., Csizmadia E., Gomperts E., Lieberum J.L., Hauser C.J., Ji X., Wang B., Camara N.O.S., Robson S.C. (2018). Carbon monoxide protects the kidney through the central circadian clock and CD39. Proc. Natl. Acad. Sci. USA.

[91] Kaur J., Kaur T., Sharma A.K., Kaur J., Yadav H.N., Pathak D., Singh A.P. (2021). Fenofibrate attenuates ischemia reperfusion-induced acute kidney injury and associated liver dysfunction in rats. Drug Dev. Res..

[92] Tanaka T., Nangaku M. (2013). Angiogenesis and hypoxia in the kidney. Nat. Rev. Nephrol..

[93] Nakada M., Kita D., Watanabe T., Hayashi Y., Teng L., Pyko I.V., Hamada J. (2011). Aberrant signaling pathways in glioma. Cancers.

[94] Meng F., Zhang Z., Chen C., Liu Y., Yuan D., Hei Z., Luo G. (2021). PI3K/AKT activation attenuates acute kidney injury following liver transplantation by inducing FoxO3a nuclear export and deacetylation. Life Sci..

[95] Sang Z., Dong S., Zhang P., Wei Y. (2021). miR-214 ameliorates sepsis-induced acute kidney injury via PTEN/AKT/mTOR-regulated autophagy. Mol. Med. Rep..

[96] Glaviano A., Foo A.S.C., Lam H.Y., Yap K.C.H., Jacot W., Jones R.H., Eng H., Nair M.G., Makvandi P., Geoerger B. (2023). PI3K/AKT/mTOR signaling transduction pathway and targeted therapies in cancer. Mol. Cancer.

[97] Wang S.S., Chen Y.H., Chen N., Wang L.J., Chen D.X., Weng H.L., Dooley S., Ding H.G. (2017). Hydrogen sulfide promotes autophagy of hepatocellular carcinoma cells through the PI3K/Akt/mTOR signaling pathway. Cell Death Dis..

[98] Benavides G.A., Squadrito G.L., Mills R.W., Patel H.D., Isbell T.S., Patel R.P., Darley-Usmar V.M., Doeller J.E., Kraus D.W. (2007). Hydrogen sulfide mediates the vasoactivity of garlic. Proc. Natl. Acad. Sci. USA.

[99] Chiba T., Oda A., Zhang Y., Pfister K.E., Bons J., Bharathi S.S., Kinoshita A., Zhang B.B., Richert A.C., Schilling B. Loss of long-chain acyl-CoA dehydrogenase protects against acute kidney injury. JCI Insight.

[100] Piko N., Bevc S., Hojs R., Ekart R. (2023). The Role of Oxidative Stress in Kidney Injury. Antioxidants.

[101] Xie C., Zhou X., Chen W., Ren D., Li X., Jiang R., Zhong C., Zhu J. (2024). Diallyl trisulfide induces pyroptosis and impairs lung CSC-like properties by activating the ROS/Caspase 1 signaling pathway. Chem. Biol. Interact..

[102] Zheng J., Cheng X., Xu S., Zhang L., Pan J., Yu H., Bao J., Lu R. (2019). Diallyl trisulfide induces G2/M cell-cycle arrest and apoptosis in anaplastic thyroid carcinoma 8505C cells. Food Funct.

[103] Majumder S., Pushpakumar S., Juin S.K., Jala V.R., Sen U. (2022). Toll-like receptor 4 mutation protects the kidney from Ang-II-induced hypertensive injury. Pharmacol. Res..

[104] Kanda J., Mori K., Kawabata H., Kuwabara T., Mori K.P., Imamaki H., Kasahara M., Yokoi H., Mizumoto C., Thoennissen N.H. (2015). An AKI biomarker lipocalin 2 in the blood derives from the kidney in renal injury but from neutrophils in normal and infected conditions. Clin. Exp. Nephrol..

[105] Zhang J., Rudemiller N.P., Patel M.B., Wei Q., Karlovich N.S., Jeffs A.D., Wu M., Sparks M.A., Privratsky J.R., Herrera M. (2016). Competing Actions of Type 1 Angiotensin II Receptors Expressed on T Lymphocytes and Kidney Epithelium during Cisplatin-Induced AKI. J. Am. Soc. Nephrol..

[106] Wang Y., Zhang Y., Shou S., Jin H. (2023). The role of IL-17 in acute kidney injury. Int. Immunopharmacol..

[107] Anders H.J. (2016). Of Inflammasomes and Alarmins: IL-1beta and IL-1alpha in Kidney Disease. J. Am. Soc. Nephrol..

[108] Gentle M.E., Shi S., Daehn I., Zhang T., Qi H., Yu L., D’Agati V.D., Schlondorff D.O., Bottinger E.P. (2013). Epithelial cell TGFbeta signaling induces acute tubular injury and interstitial inflammation. J. Am. Soc. Nephrol..

[109] Hu Z., Zhan J., Pei G., Zeng R. (2023). Depletion of macrophages with clodronate liposomes partially attenuates renal fibrosis on AKI-CKD transition. Ren. Fail..

[110] Johnson F.L., Patel N.S.A., Purvis G.S.D., Chiazza F., Chen J., Sordi R., Hache G., Merezhko V.V., Collino M., Yaqoob M.M. (2017). Inhibition of IkappaB Kinase at 24 Hours After Acute Kidney Injury Improves Recovery of Renal Function and Attenuates Fibrosis. J. Am. Heart Assoc..

[111] Ruiz S., Vardon-Bounes F., Virtos M., Seguin T., Crognier L., Rouget A., Georges B., Conil J.M., Minville V. (2023). Influence of arterial blood gases on the renal arterial resistive index in intensive care unit. J. Transl. Med..

[112] Iciek M., Bilska-Wilkosz A., Gorny M., Sokolowska-Jezewicz M., Kowalczyk-Pachel D. (2016). The Effects of Different Garlic-Derived Allyl Sulfides on Anaerobic Sulfur Metabolism in the Mouse Kidney. Antioxidants.

[113] Iciek M., Kwiecien I., Chwatko G., Sokolowska-Jezewicz M., Kowalczyk-Pachel D., Rokita H. (2012). The effects of garlic-derived sulfur compounds on cell proliferation, caspase 3 activity, thiol levels and anaerobic sulfur metabolism in human hepatoblastoma HepG2 cells. Cell Biochem. Funct..

